# Association Between Self‐Reported Potentially Modifiable Cardiac Risk Factors and Perceived Need to Improve Physical Health: A Population‐Based Study

**DOI:** 10.1161/JAHA.117.005491

**Published:** 2017-05-03

**Authors:** F. Daniel Ramirez, Yue Chen, Pietro Di Santo, Trevor Simard, Pouya Motazedian, Benjamin Hibbert

**Affiliations:** ^1^ Division of Cardiology University of Ottawa Heart Institute Ottawa Ontario Canada; ^2^ CAPITAL Research Group University of Ottawa Heart Institute Ottawa Ontario Canada; ^3^ School of Epidemiology, Public Health and Preventive Medicine University of Ottawa Ontario Canada

**Keywords:** epidemiology, lifestyle, myocardial infarction, prevention, risk factors, Cardiovascular Disease, Lifestyle, Risk Factors, Epidemiology

## Abstract

**Background:**

An individual's perceived need to improve their physical health (PNIPH) is an essential precursor to adopting healthy behaviors. Nine potentially modifiable risk factors (PMRFs) for myocardial infarction collectively account for ≥90% of the population attributable risk. Though widely recognized, their impact on individuals’ health perceptions is unclear.

**Methods and Results:**

Residents from 6 provinces were administered a module on changes to improve health as part of the 2011–2012 Canadian Community Health Survey, yielding relevant data for 8 of the 9 PMRFs sought. The potential effects of PMRFs individually and cumulatively on PNIPH were examined using modified Poisson regression. In total, 45 443 respondents were included, representing 11 006 123 individuals and corresponding to 96.8% of the adult population of the sampled provinces. The sum of PMRFs was positively associated with PNIPH (adjusted prevalence ratio, 1.08; 95% CI, 1.07–1.09 per additional PMRF) with 82.3% of individuals with ≥5 PMRFs reporting this perception. Smoking, obesity, and low physical activity were most strongly associated with PNIPH, whereas hypertension and diabetes mellitus exhibited no association with this outcome after adjusting for potential confounders. Barriers to adopting healthy behaviors were reported by 55.9% of individuals endorsing PNIPH.

**Conclusions:**

The cumulative burden of PMRFs is positively associated with PNIPH; however, individual PMRFs differentially contribute to this perception. Among those at highest cardiac risk, ≈1 in 5 denied PNIPH. A better understanding of factors underlying health perceptions and behaviors is needed to capitalize on cardiovascular preventive efforts.

## Introduction

Despite decades of steady progress, ischemic heart disease remains among the leading causes of morbidity and mortality.^1^, ^2^ Much of the progress made thus far has been attributed to advances in acute interventions and secondary preventive cardiovascular therapies; however, changes in risk factor trends are estimated to account for roughly half to two thirds of the observed improvements in developed countries.^3^, ^4^, ^5^ Emerging increases in prevalence of certain cardiovascular risk factors (particularly obesity, diabetes mellitus, and hypertension) therefore threaten to halt or potentially reverse these public health gains.^3^, ^6^ Indeed, disproportionately slower improvements or increases in mortality from ischemic heart disease among younger demographics, particularly among women, have recently been reported, collectively underscoring the need to bolster primary preventive strategies.^7^, ^8^, ^9^, ^10^, ^11^

INTERHEART identified 9 potentially modifiable risk factors (PMRFs) that collectively account for ≥90% of the population attributable risk for acute myocardial infarction (MI) worldwide: smoking, history of hypertension, diabetes mellitus, abdominal obesity, psychosocial factors, daily consumption of fruits and vegetables, regular alcohol consumption, regular physical activity, and a raised apolipoprotein (Apo)B/ApoA1 ratio. These PMRFs are associated with MI irrespective of sex or age and exhibited a cumulative effect.^12^ Though they have been well described in the medical literature and are broadly well known among laypersons, their impact on individuals’ health perceptions and behaviors is less clear. Given that an individual's perceived need to change is regarded as an essential precursor to intending and committing to behavioral changes,^13^, ^14^, ^15^ these associations have important implications for preventive health care strategies. We therefore sought to examine the association between individual and cumulative PRMFs with overall physical health perceptions using data from the 2011–2012 Canadian Community Health Survey (CCHS). We hypothesized that PMRFs would be positively associated with a perceived need to improve physical health (PNIPH) both individually and cumulatively.

## Methods

### Data Source and Sample

The 2011–2012 CCHS was a cross‐sectional survey of Canadians aged 12 years or older conducted by Statistics Canada (Ottawa, Ontario, Canada). Persons living on Indian Reserves or Crown lands, full‐time members of the Canadian Forces, institutionalized persons, and persons living in the Quebec health regions of Région du Nunavik and Région des Terres‐Cries‐de‐la‐Baie‐James were excluded from the survey (collectively accounting for <3% of potential respondents). The national combined household‐ and person‐level response rate was 68.4%.^16^ Residents from 6 provinces were administered a survey module on changes to improve health and formed the sample of our study. All analyses were restricted to individuals aged 18 years or older. Individuals who refused or otherwise did not provide an answer to the survey question relating to the outcome of interest were also excluded.

Public Use Microdata Files were used for this study, which contain anonymized data, underwent formal review and approval by an executive committee of Statistics Canada, and were made publicly available for statistical and research purposes through the Data Liberation Initiative.^16^, ^17^ Consent was obtained from all respondents at the time of survey administration.

### Variable and Outcome Definitions

Eight PMRFs were dichotomized as present or absent. Cigarette smoking was defined as current smoking or having quit smoking within the preceding 12 months. Obesity was defined by a body mass index (BMI) ≥30 kg/m^2^ as calculated from self‐reported height and weight. Low physical activity was defined as a reported mean total daily energy expenditure of <1.7 kcal/kg per day on transportation and leisure time activities, which corresponds to less than moderate exercise for 4 hours weekly (the criterion used in the INTERHEART study^12^). High stress was defined as a perceived life and/or work stress score of ≥4 on a 5‐point scale (corresponding to “quite a bit stressful” or “extremely stressful”). Hypertension was deemed present if respondents reported that they had “high blood pressure” as diagnosed by a health professional, if they were “ever diagnosed with high blood pressure” by a health professional, or if they reported having taken “any medicine for high blood pressure” in the past month. Diabetes mellitus was self‐reported as having been diagnosed by a health professional. Fruit and vegetable consumption was measured as a frequency with 5 times/day used as the cutoff.^18^, ^19^ Low or moderate alcohol intake was defined as <4 drinks/week and having consumed alcohol within the preceding 12 months.^20^ Abstinence from alcohol and ≥4 drinks/week were considered separately and as a combined variable, when appropriate. Apo levels or other measures of dyslipidemia were not included because only indirect data on this PMRF were obtained in only a subset of survey respondents.^16^

Important covariates (potential confounders or effect modifiers of the association of interest) selected a priori included age, sex, marital status, culture or racial origin, highest level of education achieved, total yearly household income, and having a regular medical doctor. Age was categorized into 3 groups: 18 to 39, 40 to 59, and ≥60 years. Marital status was classified as single, never married; married or in a common‐law relationship; or widowed, separated, or divorced. Culture or racial origin was categorized as white or visible minority. Highest level of education was grouped into 3 levels: less than secondary school graduation; secondary school graduation without postsecondary education; and some postsecondary education (with or without program completion). Total yearly household income was divided into 3 levels: <$40 000, $40 000 to $79 999, and ≥$80 000 (Canadian dollars). Canadian dollars were comparable to US dollars during the 2011–2012 period.

Data on smoking were missing in 0.7%, BMI in 3.4%, physical activity in 0.7%, stress in 1.2%, hypertension in <0.1%, diabetes mellitus in 0.1%, fruit and vegetable consumption in 5.7%, alcohol intake in 1.7%, marital status in 0.2%, culture or racial origin in 3.1%, highest education level achieved in 3.4%, household income in <0.1%, and having a regular medical doctor in <0.1%. Data were complete for age and sex. For all variables with ≥1% missing data, an additional category (“unknown”) was created; otherwise, respondents with missing data were excluded from analyses. The number of self‐reported PMRFs per respondent was calculated as their sum, each based on responses to relevant survey questions.

The outcome of interest was PNIPH as defined by an affirmative response to the question, “Do you think there is anything you should do to improve your physical health?”. Responses to follow‐up questions on specific behavior changes were used to confirm the relevance of affirmative responses to cardiac risk. Analyses of responses to the questions, “Is there anything stopping you from making this improvement?”, and “What is [stopping you from making this improvement]?” among individuals endorsing PNIPH were also undertaken to gain insight into barriers to behavioral change.

### Statistical Analyses

The CCHS uses a complex survey design with stratification, multiple stages of selection, and unequal probability sampling,^16^ which was taken into consideration in point and variance estimations.^21^ First, relative weights were calculated by dividing survey weights by average weights for responses from all respondents. Adjusted weights were then calculated by dividing relative weights by the square root of the average design effect, which is a mean of the coefficients of variation for the study variables (provided by Statistics Canada^22^). The use of this approximate method of incorporating the design effect tends to yield conservative tests of significance.^23^

Chi‐square and *t* tests were used to examine associations between all variables of interest and the outcome. The associations of individual PMRFs and their sum with the outcome were assessed by modified Poisson regression using a robust error variance procedure (sandwich estimation).^24^ Multivariable models were fit by manual forward and backward selection using the change in the exposure‐outcome association and clinical judgment to guide covariate inclusion. Adjusted prevalence ratios for combinations of PMRFs were derived by summation of their respective model coefficients.^12^ Effect measure modification was assessed by interaction terms and comparison of stratified effect measures. All analyses were performed using SAS software (version 9.4; SAS Institute Inc, Cary, NC). An alpha level of 0.05 was used to define statistical significance (2‐tailed analyses). All continuous variables are reported as mean±SD or median (interquartile range; IQR) and all categorical variables as number (%). All prevalence ratios (PRs) are provided with 95% CIs.

Sensitivity analyses were performed to evaluate the impact of missing values for variables with overall ≥1% missing data. Additionally, the data were reanalyzed using a BMI criterion of 25 kg/m^2^ (to include overweight status as a PMRF) and a cutoff for mean total daily energy expenditure of <1.1 kcal/kg per day on transportation and leisure time activities (≈150 min/week of moderate exercise) to define low physical activity as per the American Heart Association definitions for ideal cardiovascular health.^6^ Last, all prespecified analyses were repeated using data from the 2013–2014 CCHS, which sampled fewer, but different, provinces.^17^

## Results

### Respondent Characteristics and Relevance of the Outcome to Cardiac Risk

**Figure 1:**
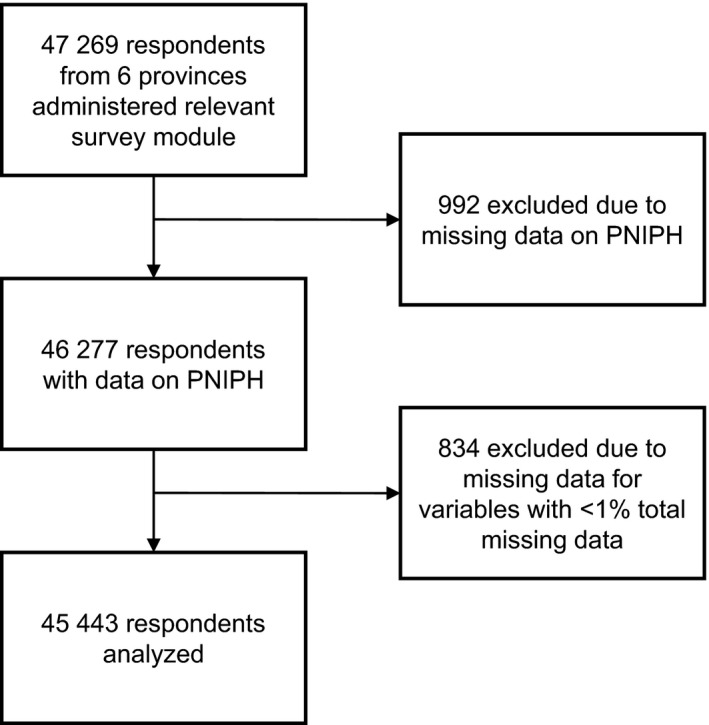
Study sample selection. PNIPH indicates perceived need to improve physical health.

A total of 45 443 respondents were included in the analyses after excluding 992 (2.1%) from whom the outcome of interest was not provided and 834 (1.8%) because of missing values for variables with <1% total missing data (Figure 1). This sample represented 11 006 123 individuals, corresponding to 96.8% of the adult population of the 6 provinces included and 40.9% of the entire country. Respondents were from Alberta (25.6%), Manitoba (8.0%), Quebec (55.4%), Nova Scotia (6.6%), Prince Edward Island (1.0%), and Newfoundland and Labrador (3.6%). The mean number of PMRFs was 2.5±1.0 with a median of 2 (IQR, 1–3). Mean BMI was 26.1±3.7 kg/m^2^.

**Figure 2:**
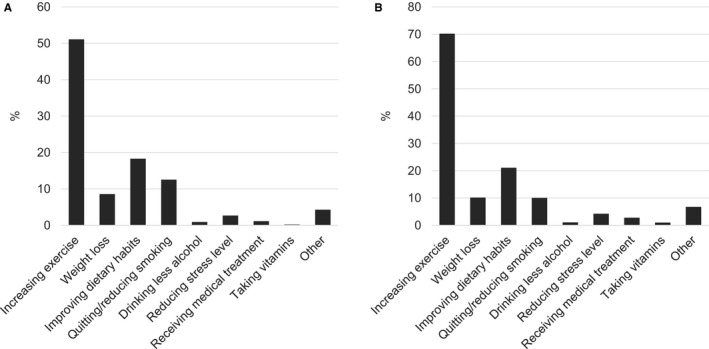
Relevance of a perceived need to improve physical health on cardiac risk. A, Most important behavioral change to improve health reported by individuals endorsing a perceived need to improve their physical health. B, Health behavior changes planned among individuals who reported intending to improve their physical health in the upcoming year.

Overall, 73.6% of individuals reported PNIPH, of which 81.1% reported an intention to improve their health in the upcoming year. Among individuals who endorsed PNIPH, 99.8% identified a specific behavioral change as being most important, of which increasing exercise, losing weight, improving dietary habits, and quitting/reducing smoking accounted for 90.7% (Figure 2A). These behaviors were also the most commonly reported changes planned among individuals who intended to improve their health within the year (Figure 2B).

### Exploratory Analyses

Level of education was positively associated with PNIPH in unadjusted analyses. In contrast, older age; being widowed, separated, or divorced; low total yearly household income; and having a regular medical doctor were associated with a reduced likelihood of this perception. Men and women expressed this opinion in comparable proportions, as did whites and those of a visible minority. Smoking, obesity, low physical activity, high stress, and low fruit and vegetable consumption were positively associated with PNIPH; however, hypertension, diabetes mellitus, and lack of or excessive alcohol intake were not. The sum of self‐reported PMRFs was nevertheless significantly associated with PNIPH (Table 1).

**Table 1:** Prevalence of PNIPH According to Respondent Demographics and PMRF

	Overall Sample	No. Reporting PNIPH	Weighted % Reporting PNIPH^a^	*P* Value^b^
Age, y				
18 to 39	13 862	10 853	78.4	<0.001
40 to 59	14 642	11 249	78.4	
≥60	16 939	9629	59.3	
Sex				
Men	19 933	13 927	73.6	0.989
Women	25 510	17 804	73.6	
Marital status				
Married/common‐law	25 311	18 190	74.4	<0.001
Widowed/separated/divorced	9621	5762	66.0	
Single, never married	10 511	7779	75.9	
Cultural or racial origin				
White	39 685	27 687	73.9	<0.001
Visible minority	4429	3274	74.3	
Unknown	1329	770	63.7	
Education				
Less than sec. school graduate	8801	5002	62.0	<0.001
Sec. school grad, no post‐sec	7448	5314	72.5	
Post‐sec. education^c^	27 715	20 580	77.0	
Unknown	1479	835	64.1	
Annual household income				
≤$39 999	16 166	9817	65.4	<0.001
$40 000 to $79 999	15 581	11 329	75.5	
≥$80 000	13 696	10 585	77.9	
Having a regular medical doctor				
Yes	37 892	26 094	73.0	<0.001
No	7551	5637	76.1	
Smoker^d^				
Yes	11 220	8973	83.0	<0.001
No	34 223	22 758	70.4	
Obesity^e^				
Yes	9356	7590	83.7	<0.001
No	34 525	23 108	71.4	
Unknown	1562	1033	71.3	
Physical activity^f^				
Low	25 211	18 506	77.7	<0.001
High	20 232	13 225	69.0	
Stress				
High	12 770	10 065	81.4	<0.001
Low	32 114	21 325	69.8	
Unknown	559	341	63.9	
Hypertension				
Yes	13 713	9066	70.5	<0.001
No	31 730	22 665	74.6	
Diabetes mellitus				
Yes	3945	2623	69.9	<0.001
No	41 498	29 108	73.9	
Fruit and vegetable consumption				
<5 times/day	25 613	18 663	76.4	<0.001
≥5 times/day	17 332	11 652	70.9	
Unknown	2498	1416	62.2	
Alcohol intake				
Abstinent	8840	5236	63.8	<0.001
Excessive (≥4 drinks/week)	5889	4200	75.9	
Low/mod. (<4 drinks/week)	30 049	21 912	75.8	
Unknown	665	383	63.3	
No. of PMRFs				
0	2706	1514	59.1	<0.001
1	8081	5131	66.8	
2	12 068	8249	72.9	
3	11 327	8230	76.7	
4	7185	5431	80.6	
≥5	4076	3176	82.3	
No. of PMRFs				
<3	22 855	14 894	69.1	<0.001
≥3	22 588	16 837	78.8	

PMRF indicates potentially modifiable cardiac risk factor; PNIPH, perceived need to improve physical health; sec., secondary.

a Weighted to the general population.

b Chi‐square test of independence between variable and PNIPH.

c With or without obtaining a postsecondary certificate/diploma or university degree.

d Defined as current smoker or having quit smoking within the preceding 12 months.

e Defined as body mass index ≥30 kg/m^2^.

f Reported mean total daily energy expenditure <1.7 kcal/kg per day on transportation and leisure time activities (approximating <4 hours of moderate exercise/week).

### Associations Between Individual and Cumulative PMRFs and PNIPH

Modified Poisson regression models for both individual and the sum number of PMRFs were fit. Multivariable models including all 8 PMRFs identified smoking, obesity, and low physical activity as most strongly associated with PNIPH. The association with the outcome was less marked for high stress and low fruit and vegetable consumption. Self‐reported hypertension, diabetes mellitus, and excessive alcohol consumption were not associated with PNIPH whereas abstinence from alcohol was negatively associated with this perception (Table 2).

**Table 2:** Unadjusted and Adjusted Prevalence Ratios for PNIPH Associated With Individual PMRF

	Unadjusted PR (95% CI)	Adjusted PR (95% CI)^a^	*P* Value^a^
Smoking	1.14 (1.11, 1.18)	1.14 (1.10, 1.18)	<0.001
Obesity	1.18 (1.14, 1.22)	1.17 (1.13, 1.22)	<0.001
Low physical activity	1.12 (1.08, 1.15)	1.13 (1.10, 1.17)	<0.001
High stress	1.14 (1.10, 1.17)	1.09 (1.05, 1.12)	<0.001
Hypertension	0.94 (0.91, 0.98)	1.03 (0.99, 1.08)	0.111
Diabetes mellitus	0.97 (0.91, 1.03)	1.04 (0.97, 1.10)	0.286
Low fruit/vegetable consumption	1.04 (1.01, 1.08)	1.06 (1.03, 1.09)	<0.001
Abstinence from alcohol^b^	0.85 (0.81, 0.97)	0.89 (0.85, 0.93)	<0.001
Excessive alcohol intake^b^	1.01 (0.96, 1.05)	1.03 (0.99, 1.08)	0.141

PR indicates prevalence ratio.

a Adjusted for age, sex, marital status, culture or racial origin, highest level of education achieved, total yearly household income, and having a regular medical doctor.

b Reference: low/moderate alcohol consumption (<4 drinks/week).

**Figure 3:**
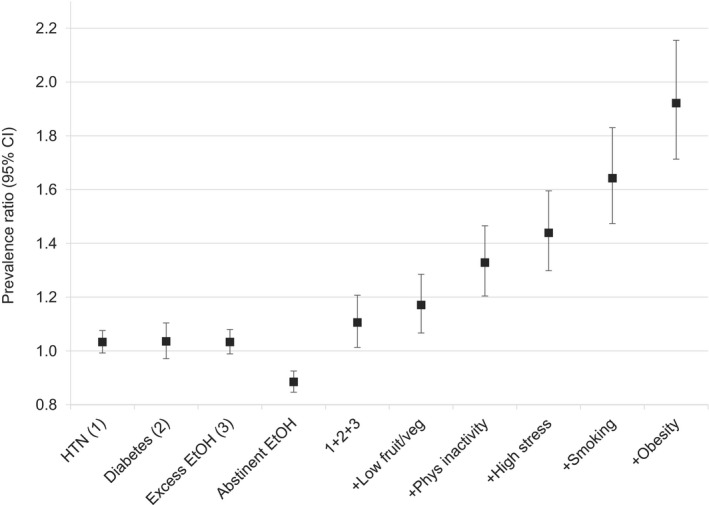
Perceived need to improve physical health associated with multiple potentially modifiable cardiac risk factors. Prevalence ratios adjusted for age, sex, marital status, culture or racial origin, highest level of education achieved, total yearly household income, and having regular medical doctor. Presented in manner analogous to INTERHEART study report.^12^ EtOH indicates alcohol; fruit/veg, fruit and vegetables; HTN, hypertension; phys, physical.

Overall, each additional PMRF was associated with a PR of 1.06 (95% CI, 1.05–1.07) for PNIPH. Controlling for potential confounders increased this PR to 1.08 (95% CI, 1.07–1.09), with all terms in the multivariable model reaching statistical significance except for culture or racial origin and having a regular doctor, which did not change the adjusted PR when removed (Table 3). All variables were kept in the final model to generate adjusted and stratified PRs because of their impact on the effect measure and/or clinical relevance. Interaction terms suggested that age, culture or racial origin, and total yearly household income modified this association (*P*_interaction_≤0.025). Older individuals and those self‐identifying as white were more likely to endorse PNIPH relative to their younger counterparts and to those self‐identifying as being of visible minorities, respectively (Table 4). Given the nonuniform contribution of individual PMRFs to PNIPH, specific combinations of PMRFs were examined (Figure 3). The combination of all PMRFs (including excessive alcohol consumption) was associated with a PR of 1.92 (95% CI, 1.71–2.16).

**Table 3:** Factors Other Than PMRF Associated With PNIPH

	PR (95% CI)	*P* Value
Age 18 to 39 y^a^	1.30 (1.24–1.36)	<0.001
Age 40 to 59 y^a^	1.26 (1.21–1.32)	<0.001
Male sex^b^	0.96 (0.94–0.99)	0.001
Married or common‐law^c^	0.99 (0.96–1.03)	0.685
Widowed, separated, or divorced^c^	0.95 (0.90–1.01)	0.008
Sec. school graduate, no post‐sec.^d^	1.11 (1.04–1.17)	<0.001
Postsecondary education^d^	1.17 (1.12–1.23)	<0.001
Education level unknown^d^	1.11 (0.95–1.28)	0.015
Visible minority^e^	0.97 (0.93–1.01)	0.086
Culture/racial origin unknown^e^	0.94 (0.81–1.10)	0.188
Household income $40 000 to $79 999/y^f^	1.10 (1.06–1.15)	<0.001
Household income ≥$80 000/y^f^	1.10 (1.06–1.15)	<0.001
Having a regular medical doctor	1.00 (0.96–1.03)	0.806

sec. indicates secondary.

a Reference: age ≥60 years.

b Reference: female sex.

c Reference: single, never married.

d Reference: less than secondary school graduation.

e Reference: self‐identified white.

f Reference: income ≤$39 999/year.

**Table 4:** Unadjusted and Adjusted Prevalence Ratios for PNIPH Associated With Each Additional PMRF and With a High Burden of PMRFs (≥3) According to Important Covariates

	PR Per PMRF (95% CI)	PR for ≥3 PMRFs (95% CI)^a^
Unadjusted	1.06 (1.05, 1.07)	1.14 (1.12, 1.16)
Adjusted^b^	1.08 (1.07, 1.09)	1.19 (1.17, 1.22)
Men	1.09 (1.07, 1.10)	1.20 (1.16, 1.24)
Women	1.08 (1.07, 1.09)	1.18 (1.15, 1.21)
Age 18 to 39 y	1.08 (1.07, 1.10)	1.18 (1.15, 1.22)
Age 40 to 59 y	1.07 (1.05, 1.08)	1.15 (1.12, 1.19)
Age ≥60 y	1.10 (1.09, 1.12)	1.27 (1.21, 1.32)
White	1.09 (1.08, 1.10)	1.20 (1.18, 1.23)
Visible minority	1.06 (1.03, 1.09)	1.13 (1.06, 1.21)
Income ≤$39 999/y	1.08 (1.06, 1.09)	1.21 (1.16, 1.27)
Income $40 000 to $79 999/y	1.07 (1.06, 1.09)	1.16 (1.13, 1.20)
Income ≥$80 000/y	1.09 (1.08, 1.10)	1.20 (1.16, 1.23)

*P*<0.001 for all PRs. PMRF indicates potentially modifiable cardiac risk factor; PR, prevalence ratio.

a Reference: <3 PMRFs.

b Adjusted for age, sex, marital status, culture or racial origin, highest level of education achieved, total yearly household income, and having a regular medical doctor.

### Perceived Barriers to Improving Physical Health

**Figure 4:**
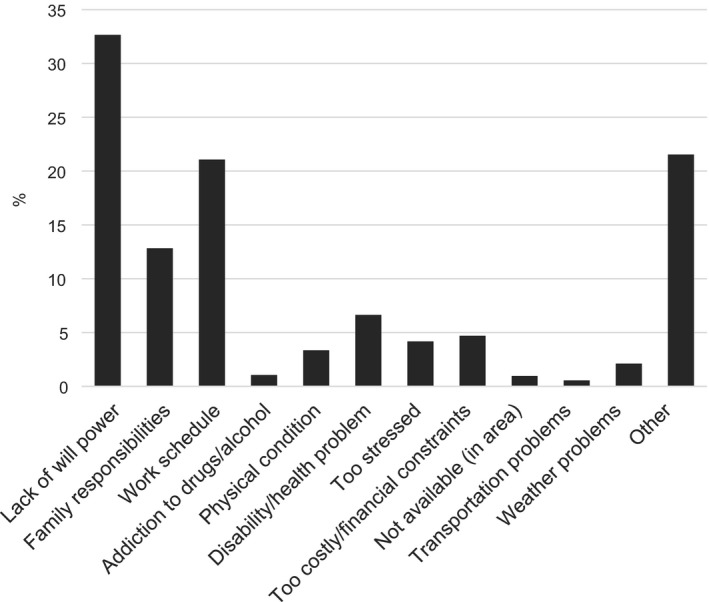
Perceived barriers to adopting positive health behaviors among individuals endorsing a perceived need to improve their physical health. Respondents could identify more than 1 barrier (mean, 1.1±0.4; range, 1–7).

Barriers to adopting positive health changes were reported by 55.9% of individuals with PNIPH. The most frequently cited barriers were a lack of will power or self‐discipline, work schedule, and family responsibilities. Cost, stress, lack of available resources in an individual's area, and problems with transportation were each identified by fewer than 5% (Figure 4).

### Sensitivity Analyses

Excluding all respondents with missing data for any PMRF or demographic variable yielded a sample size of 39 772. Unadjusted and adjusted PRs for individual PMRFs were comparable with the preceding analyses, with all point estimates remaining within 0.01 of those presented in Table 2. Similarly, point estimates of the unadjusted, overall adjusted, and strata‐specific adjusted effect measures for the sum of PMRFs and for a high burden of PMRFs (≥3) remained within 0.01 and 0.02 of those presented in Table 4, respectively. Redefining low physical activity as mean total daily energy expenditure of <1.1 kcal/kg per day on transportation and leisure time activities and differentiating normal weight from overweight/obesity by using a BMI cutoff of 25 kg/m^2^ resulted in an additional 2626 (5.8%) respondents having ≥3 PMRFs. The weighted proportions of individuals with ≥3 and ≥5 PMRFs reporting PNIPH were similar at 78.5% and 80.1%, respectively, however. Adjusted PRs of individual PMRFs for the outcome of interest remained within 0.02 of those presented in Table 2. Similarly, the point estimates of adjusted and strata‐specific PRs for the sum of PMRFs and for a high burden of PMRFs (≥3) again remained within 0.01 and 0.02 of those presented in Table 4, respectively.

The 2013–2014 CCHS had a national combined household‐ and person‐level response rate of 66.2%.^17^ It administered the relevant survey module on changes to improve health to 4 provinces: British Columbia, Manitoba, Nova Scotia, and Prince Edward Island, resulting in a smaller sample size (n=26 315) after excluding those in whom the outcome of interest was not provided and with missing values for variables with <1% total missing data. This represented 5 227 803 individuals, which is 47.5% of the population that was covered in the 2011–2012 version used in our main analyses. The mean number of PMRFs was 2.4±1.0 with a median of 2 (IQR, 1–3). Mean BMI was 26.1±3.8 kg/m^2^. Weighted associations between respondent demographics or PMRFs and PNIPH were comparable to those identified in the 2011–2012 CCHS (Table S1). Weighted crude and adjusted PRs for individual PMRFs as well as for the sum of PMRFs and a high burden of PMRFs were also similar (Tables 5 and 6).

**Table 5:** Unadjusted and Adjusted Prevalence Ratios for PNIPH Associated With Individual PMRFs From the 2013–2014 Canadian Community Health Survey

	Unadjusted PR (95% CI)	Adjusted PR (95% CI)^a^	*P* Value^a^
Smoking	1.15 (1.10, 1.20)	1.16 (1.11, 1.22)	<0.001
Obesity	1.21 (1.15, 1.27)	1.20 (1.14, 1.26)	<0.001
Low physical activity	1.10 (1.05, 1.14)	1.12 (1.07, 1.16)	<0.001
High stress	1.15 (1.10, 1.20)	1.11 (1.06, 1.16)	<0.001
Hypertension	0.92 (0.88, 0.97)	1.00 (0.95, 1.05)	0.903
Diabetes mellitus	0.96 (0.88, 1.05)	1.03 (0.94, 1.12)	0.530
Low fruit/vegetable consumption	1.07 (1.03, 1.12)	1.08 (1.04, 1.13)	<0.001
Abstinence from alcohol^b^	0.86 (0.81, 0.90)	0.90 (0.85, 0.95)	<0.001
Excessive alcohol intake^b^	1.01 (0.95, 1.07)	1.04 (0.98, 1.10)	0.239

PR indicates prevalence ratio.

a Adjusted for age, sex, marital status, culture or racial origin, highest level of education achieved, total yearly household income, and having a regular medical doctor.

b Reference: low/moderate alcohol consumption (<4 drinks/week).

**Table 6:** Unadjusted and Adjusted Prevalence Ratios for PNIPH Associated With Each Additional PMRF and With a High Burden of PMRFs (≥3) According to Important Covariates From the 2013–2014 Canadian Community Health Survey

	PR Per PMRF (95% CI)	PR for ≥3 PMRFs (95% CI)^a^
Unadjusted	1.06 (1.05, 1.07)	1.13 (1.10, 1.16)
Adjusted^b^	1.09 (1.08, 1.10)	1.20 (1.16, 1.23)
Men	1.08 (1.07, 1.10)	1.18 (1.13, 1.23)
Women	1.09 (1.08, 1.10)	1.21 (1.16, 1.26)
Age 18 to 39 y	1.10 (1.08, 1.11)	1.19 (1.14, 1.23)
Age 40 to 59 y	1.08 (1.06, 1.10)	1.19 (1.13, 1.26)
Age ≥60 y	1.09 (1.07, 1.11)	1.22 (1.17, 1.29)
White	1.09 (1.08, 1.10)	1.21 (1.18, 1.25)
Visible minority	1.08 (1.05, 1.11)	1.14 (1.06, 1.23)
Income ≤$39 999/y	1.10 (1.08, 1.13)	1.22 (1.14, 1.31)
Income $40 000 to $79 999/y	1.08 (1.06, 1.10)	1.17 (1.11, 1.24)
Income ≥$80 000/y	1.08 (1.07, 1.10)	1.20 (1.15, 1.24)

*P*<0.001 for all PRs. PMRF indicates potentially modifiable cardiac risk factor; PR, prevalence ratio.

a Reference: <3 PMRFs.

b Adjusted for age, sex, marital status, culture or racial origin, highest level of education achieved, total yearly household income, and having a regular medical doctor.

## Discussion

Ischemic heart disease is a leading cause of morbidity and mortality in North America and abroad, yet it is largely preventable with most of the risk attributed to a small number of potentially modifiable, lifestyle‐related factors.^12^ Though the importance of primary preventive strategies is well recognized, little is known about the association between PMRFs and individuals’ health perceptions and behaviors. Our study suggests (1) that the burden of PMRFs is positively associated with PNIPH, but (2) that individual PMRFs unequally contribute to this perception, and (3) that even among those at highest risk (those with ≥5 PMRFs), nearly 1 in 5 do not feel that they need to improve their physical health.

Among the PMRFs examined, smoking, obesity, and low physical activity were most strongly associated with PNIPH. Commensurate with these associations, the population attributable risks for MI of these PMRFs are substantial, reportedly ranging from 26.1% to 59.5% for North American men and women.^12^ Low fruit and vegetable consumption and high levels of stress were modestly associated with PNIPH, whereas this association was absent for diabetes mellitus, hypertension, and excessive alcohol consumption and was negative for abstinence from alcohol. Similar discrepancies in health perceptions associated with cardiovascular risk factors have previously been reported by Vähäsarja et al in their study of Finnish individuals at high risk of type 2 diabetes mellitus, finding that larger waist circumference and low physical activity were associated with a perceived need to increase physical activity levels, but that smoking, hypertension, dyslipidemia, or a family history of diabetes mellitus were not.^13^

Several points are worth noting when interpreting our results and their implications. Though beliefs about potential harms (ie, risk perceptions) play a fundamental role in shaping health behaviors,^25^ the relationship is complex^26^ and may be influenced by individual dimensions of the perceived risk (perceived likelihood, susceptibility, or severity), ease or cost of carrying out the behavior,^25^ the value ascribed to the outcome of the behavior,^13^ and sociocultural norms or attitudes.^27^ For instance, though both smoking and obesity were most strongly associated with PNIPH, these associations have not equally translated into positive behavioral changes: A continuous decline in the prevalence of smoking has been observed in North America,^28^ whereas the prevalence of obesity remains markedly high,^29^ may be increasing,^28^ and is projected to increase further.^30^ The implications of these latter trends are considerable given that elevated BMI is associated with further weight gain over the long term, compounding its associated risk.^31^ It is likely that such disparities are a product of the above influences on the risk perception/health behavior relationship. In effect, PNIPH is essential, but alone may not be sufficient to bring about health behavior change.^32^, ^33^ This is consistent with the psychological theories of behavioral change, including the theory of planned behavior, the transtheoretical model of change, and principles underlying motivational interviewing.^13^, ^14^, ^15^ It is also supported by smaller studies examining the association between PNIPH and specific intended health changes relevant to cardiac risk.^34^, ^35^, ^36^ In our study, nearly all individuals endorsing PNIPH also identified a health behavior change that was perceived as important to improving their health; however, nearly 19% reported that they did not intend to improve their health within the following year. Furthermore, though more than one half of individuals with PNIPH reported that barriers to adopting positive behavior changes existed, the most frequently cited barrier was a lack of willpower or self‐discipline.

Current North American guidelines on physical activity recommend considerably less exercise than the cutoff used in the INTERHEART study (150 versus 240 minutes of moderate exercise weekly).^6^, ^37^ Using this less‐demanding criterion for high physical activity resulted in an additional 13.4% being categorized as lacking this PMRF, but did not appreciably change its effect measure for the outcome of interest, suggesting that physical health perceptions in this group were comparable to those of individuals exercising more intensely and/or frequently. In fact, though increasing amounts of exercise generally confer increasing health benefits, most cardiovascular gains occur with at least 150 minutes—a finding that has formed the basis of current recommendations.^6^

Though fruit and vegetable consumption is known to confer cardiovascular benefits^18^, ^19^ and pertinent dietary recommendations exist,^38^, ^39^ quantifying the relationship between amount consumed and cardiovascular benefit is difficult, in part because fruits and vegetables typically form only part of an individual's diet.^6^ Cereals, meats, dairy products, and the fat and glycemic content of foods selected can contribute to cardiovascular risk or benefit.^40^, ^41^, ^42^, ^43^, ^44^ Therefore, though a diet rich in fruits and vegetables is more likely to be cardioprotective and reflective of more health‐conscious behavior, this association is imperfect. Similarly, though life and work stress were combined into 1 PMRF in our study (analogous to the home, work, and financial stress components of the combined psychosocial index used in the INTERHEART study^12^), data on other components of that index (depression, locus of control, and stressful life events)^45^ were incomplete and therefore not incorporated, potentially resulting in an underestimation of the prevalence and in a less‐precise estimate of the effect of the more broadly defined psychosocial PMRF. As well, data on respondents’ medication regimens (particularly antihypertensive and glycemic agents) or treatment effectiveness were not available or deemed sufficiently reliable to include in the study. Though self‐reported diabetes mellitus has been validated in the INTERHEART modifiable risk score model for MI,^46^ the inability to adjust for this potential confounder may account for the respective effect measures observed given that individuals being treated for hypertension or diabetes mellitus may have felt that these conditions were controlled. This could have led to under‐reporting of either or both conditions while still perceiving a need to improve physical health or incorrectly reporting the conditions, but perceiving that their health impacts were minimized. Notably, however, the importance of lifestyle modification irrespective of pharmacological treatment has been emphasized, particularly for diabetes mellitus given a lack of evidence that glycemic control alone improves macrovascular outcomes.^47^ Last, though low or moderate alcohol consumption has been associated with cardiovascular benefits,^48^ the link is complicated^20^, ^49^ and the benefits of alcohol are offset by its well‐known potential harms, which may affect its perceived health impact. Differing interpretations or estimations of this balance of risk and benefit may account for the negative association between abstinence from alcohol and PNIPH as well as for the lack of association between excessive alcohol consumption and PNIPH.

The sum of PMRFs was positively and significantly associated with PNIPH; however, individual PMRFs differentially contributed to this perception, with some not contributing at all. Though these associations may suggest a degree of public awareness of the health implications of PMRFs in general, our study suggests that it is modest and inconsistent, with a sizeable proportion of the public reporting that they do not feel that they should improve their health even among those at highest cardiovascular risk. Moreover, this association was attenuated among younger age groups and persons identifying as being of visible minorities—findings that warrant further investigation. Numerous explanations may account for these findings, including increased contact with health care providers at older ages and cultural influences on health and/or health care use^50^; however, these remain speculative given the limitations of the data set. The statistical power afforded by our sample size may have also identified minimal differences in certain cases (as in our exploratory analysis). A greater understanding of factors underlying health perceptions and behaviors, including among these subgroups, may yield considerable benefits.

There are several important limitations of our study. The outcome variable selected is inherently imperfect and likely failed to capture important nuances in health perceptions. However, perceived need to change cardiovascular health behavior has been assessed in analogous fashions by others given a lack of accepted measure for this or similar latent variables.^13^, ^34^, ^35^, ^36^ Furthermore, among those with PNIPH as defined by this variable, nearly all identified a specific lifestyle change as being most important for improving their health, the majority reported an intention to improve their physical health in the next year, and nearly all the changes planned are known to modify cardiac risk, arguing for the value of the outcome selected. Nevertheless, a panel of questions targeting different aspects of health perceptions and determinants of lifestyle behavioral change (eg, the validated Determinants of Lifestyle Behavior Questionnaire^51^) would have allowed for a more‐robust analysis. Our regression models examining the relationship between the overall sum of PMRFs and PNIPH assume that each PMRF contributes uniformly to the outcome, which we show to not be the case and which render our effect estimates less precise. However, this analysis is relevant to clinical practice given that cumulative PMRFs elicit greatest concern. Data on individual and specific combinations of PMRFs are also provided. Data were collected by self‐reports and are therefore subject to measurement error, particularly recall bias. Standardized interviewer questionnaires and the sampling strategy used^16^ render interviewer and selection bias less likely. In addition, as mentioned above, only 8 of the 9 PMRFs identified in the INTERHEART study were assessed because a robust measure of dyslipidemia was not collected. Given the strong association between dyslipidemia and other PMRFs,^52^, ^53^ it is likely that its inclusion would have attenuated the exposure‐outcome associations identified. Abdominal obesity was not included in the survey; therefore, BMI was used as a surrogate measure, which may not optimally reflect the importance of fat distribution on cardiovascular risk.^54^, ^55^ However, BMI is recommended and routinely used to detect and monitor weight in clinical practice.^56^, ^57^ Knowledge regarding additional comorbidities that may independently influence individuals’ opinions on the need to improve their physical health were unaccounted for. Last, culture or racial origin was grouped into white, visible minority, and not stated, which limits detailed analyses of potential sociocultural influences on health perceptions.

## Conclusions

The cumulative burden of PMRFs is positively associated with PNIPH; however, individually, PMRFs are differentially associated with this perception. A substantial proportion of individuals at risk for cardiovascular events do not feel a need to improve their physical health, indicating an urgent need to identify means to modify public health perceptions and behaviors.

## Disclosures

None.

## Supporting information


**Table S1.** Prevalence of Perceiving a Need to Improve Physical Health According to Respondent Demographics and Potentially Modifiable Cardiac Risk Factors From the 2013–2014 Canadian Community Health Survey
