# Supplementary material for: Association Between Self‐Reported Potentially Modifiable Cardiac Risk Factors and Perceived Need to Improve Physical Health: A Population‐Based Study
Source: J Am Heart Assoc. 2017 May 3;6(5):e005491. doi: 10.1161/JAHA.117.005491 (PMC5524095; doi:10.1161/JAHA.117.005491)
Supplement: Supplementary file 1 — Table S1. Prevalence of Perceiving a Need to Improve Physical Health According to Respondent Demographics and Potentially Modifiable Cardiac Risk Factors From the 2013–2014 Canadian Community Health Survey [file JAH3-6-e005491-s001.pdf]

SUPPLEMENTAL MATERIAL

Table S1. Prevalence of perceiving a need to improve physical health according to respondent demographics and potentially modifiable cardiac risk factors from the 2013-2014 Canadian Community Health Survey

	Overall sample	Number reporting PNIPH	Weighted % reporting PNIPH*	*P* value[†]
Age, y				
18-39	6440	4854	77.7	<0.001
40-59	7845	5889	74.6	
≥60	12 030	6996	60.5	
Sex				
Men	11 438	7651	72.2	0.267
Women	14 877	10 088	71.4	
Marital status				
Married/common-law	14 434	10 018	72.6	<0.001
Widowed/separated/divorced	6269	3685	62.6	
Single, never married	5612	4036	74.7	
Cultural or racial origin				
White	21 717	14 606	72.5	<0.001
Visible minority	4018	2806	70.8	
Unknown	580	327	60.8	
Education				
Less than sec. school grad	4189	2307	60.2	<0.001
Sec. school grad, no post-sec	5802	3899	71.3	
Post-sec. education[‡]	15 949	11 286	73.9	
Unknown	375	247	72.4	
Annual household income				
≤$39,999	9119	5491	65.5	<0.001
$40,000-$79,999	8924	6144	70.3	
≥$80,000	8272	6104	77.2	
Having a regular medical doctor				
Yes	23 398	15 602	70.7	<0.001
No	2917	2137	77.9	
Smoker[§]				
Yes	5190	4103	82.0	<0.001
No	21 125	13 636	69.3	
Obesity[‖]				
Yes	5460	4378	84.0	<0.001
No	19 899	12 741	69.1	
Unknown	956	620	68.3	
Physical activity[#]				
Low	13 042	9338	76.2	<0.001
High	13 273	8401	68.0	

Stress				
High	6505	4981	79.9	<0.001
Low	19 527	12 598	68.5	
Unknown	283	160	58.3	
Hypertension				
Yes	8735	5618	67.7	<0.001
No	17 580	12 121	73.1	
Diabetes				
Yes	2426	1588	69.0	0.057
No	23 889	16 151	72.0	
Fruit and vegetable consumption				
<5 times/day	15 376	10 826	75.0	<0.001
≥5 times/day	9344	6064	67.7	
Unknown	1595	849	58.7	
Alcohol intake				
Abstinent	5905	3409	62.5	<0.001
Excessive (≥4 drinks/week)	3704	2540	73.4	
Low/mod. (<4 drinks/week)	16 422	11 626	74.5	
Unknown	284	164	61.5	
No. of PMRFs				
0	1815	1002	57.2	<0.001
1	4787	2932	66.4	
2	6978	4585	71.7	
3	6413	4533	74.3	
4	3985	2926	79.1	
≥5	2337	1761	80.9	
No. of PMRFs				
<3	13 580	8519	67.9	<0.001
≥3	12 735	9220	76.8	

PMRF: potentially modifiable cardiac risk factor; PNIPH: perceived need to improve physical health

* Weighted to the general population

† Chi-square test of independence between variable and PNIPH

‡ With or without obtaining a post-secondary certificate/diploma or university degree

§ Defined as current smoker or having quit smoking within the preceding 12 months

|| Defined as body mass index ≥30 kg/m^2

Reported mean total daily energy expenditure <1.7 kcal/kg/day on transportation and leisure time activities (approximating <4 hours of moderate exercise/week)
